# Active Commute in Relation to Cognition and Academic Achievement in Children and Adolescents: A Systematic Review and Future Recommendations

**DOI:** 10.3390/ijerph16245103

**Published:** 2019-12-13

**Authors:** Madhura Phansikar, Sadia Anjum Ashrafi, Naiman A. Khan, William V. Massey, Sean P. Mullen

**Affiliations:** 1 Department of Kinesiology and Community Health, University of Illinois at Urbana-Champaign, Champaign, IL 61820, USA; map7@illinois.edu (M.P.); ashrafi3@illinois.edu (S.A.A.); nakhan2@illinois.edu (N.A.K.); 2 College of Public Health and Human Sciences, Oregon State University, Corvallis, OR 97331, USA; william.massey@oregonstate.edu; 3 Beckman Institute for Advanced Science and Technology, University of Illinois at Urbana-Champaign, Urbana, IL 61801, USA; 4 Illinois Informatics Institute, University of Illinois at Urbana-Champaign, Champaign, IL 61820, USA

**Keywords:** active travel, walking, bicycling, executive function

## Abstract

Active commuting to school (ACS) is an important source of physical activity among children. Recent research has focused on ACS and its benefits on cognition and academic achievement (AA), factors important for success in school. This review aims to synthesize literature on the relationship between ACS and cognition or AA among children and adolescents. Peer-reviewed articles in PubMed, Web of Science, PsycINFO and Cochrane Library assessing ACS with cognition and/or AA among children, until February 2019, were selected. Twelve studies across nine countries (age range 4–18.5 years) were included. One study used accelerometers, whereas all others used self-report measures of ACS. A wide range of objective assessments of cognitive functioning and AA domains were used. Five among eight studies, and four among six found a positive relationship between ACS and cognitive or AA measure, respectively. Four studies found dose–response relationships, and some studies found sex differences. The quantitative analysis found that ACS was not significantly associated with mathematics score (odds ratio = 1.18; CI = 0.40, 3.48). Findings are discussed in terms of methodological issues, potential confounders, and the strength of the evidence. Future studies should conduct longitudinal studies and use objective measures of ACS to understand this relationship further.

## 1. Introduction

Physical activity is important for children’s overall physical, psychosocial, and cognitive development [1]. Research in recent decades has shown that engaging in physical activity improves cognitive performance and academic achievement in children and adolescents [2,3]. Cognitive ability and academic achievement are closely linked. Indeed, improvement in academic achievement could be potentially a result of improvement in executive functioning, which is a set of higher-order functions underlying aspects such as memory, inhibition, planning, and scheduling [4]. The improvements in cognition may be partially due to structural and functional changes in the brain as a result of physical activity. Among children, leisure time physical activity (LTPA) is associated with an increase in executive functioning and associated brain structures and functions [5,6,7]. On the other hand, some studies have shown that engaging in LTPA is not predictive of cognitive functioning. It may be that aerobic fitness, rather than LTPA, is related to aspects of cognition. Since brain development occurs throughout childhood [8], it may be especially important for children to engage in physical activity to increase aerobic fitness and reap its cognitive benefits, directly or indirectly.

Although the benefits of physical activity are widely recognized, the rates of physical activity participation among children are low in the United States (US) as well as much of the world [9,10]. According to the Center for Disease Control and Prevention only one-quarter (24.8%) of the children aged 12–15 in the US met the physical activity guidelines, i.e., 60 min of moderate to vigorous physical activity (MVPA) daily, and 61.50% of children aged 9–13 years did not participate in organized LTPA, such as sports [11]. The estimates provided through objectively measured physical activity data are more dismal. According to data collected from a representative sample of the US, through the National Health and Nutritional Examination Survey, 42% of children from 6–11 years old met the physical activity guidelines, and only 8% of the adolescents met these guidelines [12]. Recent research suggests that the estimated range of children meeting the physical activity guidelines, measured using accelerometry, depends on the cut-off points used and can be as wide as 8% to 96% [13]. Around the globe, the Global Matrix of Grades comparing physical activity levels across 15 countries reported that 10 countries received a grade of D (20.00%–39.00% children meeting PA guidelines) or F (less than 20.00% meeting PA guidelines) [14]. Furthermore, the rates of obesity in this age group are on the rise [15]. Considering these trends, the CDC has called for innovative approaches that could increase physical activity levels among children. Given that the rates of LTPA are very low among children, promoting physical activity through other means, for example, active commuting to school, may be effective for increasing physical activity levels, and thereby affecting cognitive development. Active commuting involves using non-motorized modes of transport, such as walking and bicycling, whereas passive commuting involves motorized transport (e.g., cars, bus, etc.).

Active commuting to school (ACS) is categorized herein as a form of LTPA complementary to sport and chores. It is worth noting, however, that a defining attribute of LTPA is that it is discretionary, but children do not always have a choice in their mode of transportation. Still, ACS may be an important modifiable behavior that is often overlooked as a source of physical activity for children [16]. The prevalence of ACS has declined from 48% in 1969 to less than 16% in 2001 [17]. Evidence for the role of active commuting has been derived from systematic reviews of interventions promoting ACS among children and adolescents over the past decade [18,19,20]. Interventions designed to promote ACS were varied, and included improving road safety to promote walking, designing walk-to-school programs supervised by parents, providing educational resources to parents and children to promote walking to school, conducting educational sessions in the classroom, and promoting friendly competition among schoolchildren to walk to school. Most of the studies included in the review reported a small increase in the active commuting level of the participants, ranging from 3.00% to 64.00%. Other systematic reviews have reported similar findings—that is, engaging in active commuting is associated with an increase in overall physical activity level in more than 50.00% of the studies reviewed [21,22,23]. This increase was reported as an average of 28 min daily [21], and between 0 and 45 min daily [22]. Thus, ACS may be an important means by which to increase physical activity up to 60 min daily, among child and adolescent populations. Considering that LTPA has shown beneficial effects on cognition, it is possible that physical activity accumulated through other means, such as ACS, may also yield cognitive benefits through similar and other mechanisms. While ACS involves similar movements to other physical activities, as well as opportunities for social interaction, it may be unique in terms of some of its inherent cognitive-motor challenges. While it is possible for any environment encircling a child’s journey to school to serve as a stressor, it could just as easily provide a natural outlet for psychosocial stress. ACS demands attentive navigation through outdoor life-space which includes distractions and dangers (pedestrians, traffic) and yet this can, in theory, facilitate skill-development. Also, being physically active outside could improve a child’s mental health and well-being [24]. Thus, potential ACS benefits for cognitive functioning may be shared by other physical activities (whereby ACS would simply provide a greater dose), yet there may be some benefits unique to ACS.

Previous systematic reviews have shown that ACS has a positive impact on body composition [25], cardiorespiratory and cardiovascular fitness [22,25], and total physical activity levels, with insufficient evidence for impact on body weight [21,22,23,26]. In recent years, there has been growing research on the cognitive benefits of ACS, among children and adolescents. A number of studies have looked at the association between walking and/or bicycling to school, and cognitive improvement or academic achievement. However, there is no systematic review that has synthesized the findings of these studies. Therefore, the purpose of this study was to conduct a systematic review of studies done with children and adolescents, investigating the relationship between ACS, and cognitive functioning or academic achievement. We have focused on exploring this relationship among adolescents, to improve the interpretation of homogenous comparisons across studies.

## 2. Materials and Methods

A systematic review and quantitative analysis were conducted in accordance with the Preferred Reporting Items for Systematic Reviews and Meta-Analyses (PRISMA) [27]. The PRISMA checklist is included in Appendix A Table A1.

### 2.1. Study Selection Criteria

Studies meeting the following eligibility criteria were included in the systematic review: (1) Study designs: randomized control trial, pre–post study, longitudinal study, cross-sectional study, and case control study; (2) Population: children and adolescents (17 years of age and younger); (3) Exposure: active commuting; (4) Outcome: cognitive function and/or academic achievement; (5) Publication: peer reviewed journal article; (6) Time window of search: from inception of an electronic bibliographic database to 23 February 2019; and (7) Language: English.

Studies were excluded if they met any of the following criteria: (1) Studies with adult participants (18+ years of age); (2) Studies not measuring active travel but rather other types of physical activity, such as other types of LTPA (e.g., playing baseball), physical education class, or occupational physical activity; (3) Studies that do not specifically assess the association between active commuting and concerned outcomes; (4) Studies that do not differentiate between active commuting and other types of physical activity and/or other modes of commuting; (5) Cognitive outcomes related to mental health dysfunction, such as mild cognitive impairment and autism; (6) Non-English publication; (7) review or case study; and (8) Non-peer reviewed article (e.g., dissertation or conference proceeding).

### 2.2. Search Strategy

Keyword search was conducted in the PubMed, Cochrane Library, PsycINFO, and Web of Science core collection. These databases were selected for their relevancy and because their sheer size and access to scientific articles offered the greatest likelihood of capturing available literature consistent with our study’s scope. The search terms used were a combination of synonyms related to active commuting, cognition, academic achievement, and children and adolescents. The search algorithm used for each database can be found in Appendix B. Two authors (M.P. and S.A.) conducted the title and abstract screening of the articles identified through the keyword search, against the study selection criteria, independently. Potentially relevant articles were identified for full-text evaluation, which was conducted independently by the two authors. Interrater reliability, calculated using Kappa co-efficient, was 0.85. A cited reference search (i.e., forward reference search) and reference list search (i.e., backward reference search) were conducted on the articles identified for inclusion following the full-text review. Relevant articles identified were evaluated against the study selection criteria and subject to further reference search, until no new relevant articles were found. Additionally, a manual search was conducted through Google Scholar. M.P. and S.A. jointly determined the studies to be included in the review following full-text reading, and discrepancies were resolved through their discussions.

### 2.3. Data Extraction and Synthesis

A standard data extraction form was used to retrieve demographic, methodological, and exposure and outcomes variables from each of the included studies and was cataloged using Microsoft word. This included first author and publication year, study design and setting, statistical model, sample characteristics (n, age, gender), features of active commuting (type, duration, measurement), measures and domain of cognitive outcome and academic achievement, and the estimated relationship between active commuting and the outcomes (shown in Table 1, Table 2 and Table 3).

### 2.4. Quantitative Analysis

A quantitative analysis was conducted to determine the pooled effect of engaging in ACS on the mathematics performance domain of academic achievement, in terms of odds ratio. Math performance was measured by the grade on the math test administered as part of the school curriculum. Among the six studies that measured math performance, only two were included in the analysis due to variation in outcome measurement and statistical technique. Study heterogeneity was assessed using the *I*^2^ index, and it was determined to be considerable (*I*^2^ > 85.00%) as well as significant (*p* < 0.05). Since study heterogeneity was high, a random effects model was used. Publication bias was not assessed due to a small number of studies in the analysis. All statistical analyses were conducted by using the Stata 14.2 SE version (StataCorp, College Station, TX, USA). All analyses used two-sided tests, and p-values less than 0.05 were considered statistically significant.

### 2.5. Study Quality Assessment

There is a lack of consensus on which study quality assessment metrics should be used [28], and whether they offer any meaningful metrics [29]. As such, caution should be taken before judging “high-quality” vs. “low-quality” evidence, just as caution should be taken before drawing any clear conclusions from the limited data available and retrieved from the scope of studies targeted. Quality assessments are limited to what has been reported and presence of certain qualities are quantified for descriptive purposes. We have tallied the presence of certain study qualities using the checklist developed by National Heart, Lung and Blood Institute, for assessment of observational, cohort, and cross-sectional studies [30].

## 3. Results

### 3.1. Study Selection

**Figure 1:**
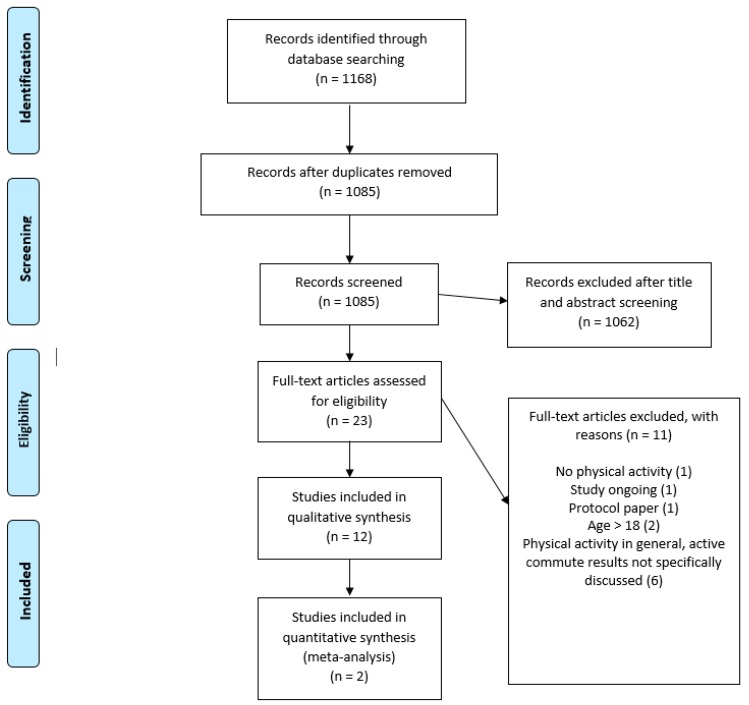
Study selection flowchart.

The study selection flowchart is shown in Figure 1. We identified a total of 1168 articles using the keyword search. After removing duplicates (n = 83), we screened 1085 articles for eligibility using title and abstract screening. A total of 1062 records were excluded, as they did not fit our inclusion criteria. Specifically, studies were excluded at this stage if they focused on physical activities conducted after school or during recess, or targeted perceptions of safety when commuting to school. A total of 23 articles were shortlisted for full-text review. After a thorough screening of these articles against the review protocol, 12 articles were included in the final synthesis. One study had an age range of 13–18.5 years, which slightly violated the inclusion criteria of below 18 years. However, we decided to retain this study because the mean age of the sample was 15.4 years, with a standard deviation of 1.3. One study that met all of our inclusion criteria was excluded because it was a protocol/methods article [31]. A forward and backward search was conducted, and no new articles were identified for inclusion. Therefore, 12 articles were included in the systematic review. The reasons for exclusion of studies were: physical activity measured in general and active commute results not specifically discussed [32,33,34,35,36,37]; age >18 [38,39]; no physical activity [40]; study currently ongoing (protocol published in clinical trials) [41]; and study protocol [31]. Figure 1 shows the study selection flowchart.

### 3.2. Summary of the Selected Studies

Table 1 provides a summary of basic characteristics of the studies included in the review and the details of study design. Studies were conducted across nine countries, including Spain (n = 4), Sweden (n = 1), Finland (n = 1), Norway (n = 1), Netherlands (n = 1), Denmark (n = 1), Taiwan (n = 1), Chile (n = 1), and Israel (n = 1). Studies were published between 2011 and 2018. The sample sizes varied substantially across studies, ranging from 92 participants [42] to 2897 participants [43]. The sample had children and adolescents across primary and secondary schools, ranging from grades 1 to 10, and age ranging from 4 to 18.5 years. Approximately 50.00% of the studies focused on pre-adolescents. All of the studies had approximately 50.00% girls as a part of their sample, with the lowest and the highest percentage being 42.47% [44] and 53.26% [42], respectively. Most of the studies (n = 10) were cross-sectional studies [42,45,46,47,48,49,50,51,52,53], and two were prospective studies [43,44]. Eight studies accounted for socioeconomic status either by asking their socioeconomic status or by using parental education as a proxy for it. Six studies reported a majority of the participants being a middle-to-high socioeconomic group or college level parental education. In one study, a majority of the participants’ parents had vocational training or <3 years of formal education [48], and another reported a majority of the parents having polytechnic education level [44]. 

Table 2 provides a summary of the characteristics of active commuting, and measurement of active commuting, cognition and academic achievement. In regard to types of active commuting, one study measured only walking [50], one study did not clarify the type included [43], and all other studies (n = 10) included both walking and cycling. Among these ten studies, one study included inline skates and scooters as constituting cycling [53]. All studies measured active commuting in the context of commuting to school; either to, from or both. Seven studies [43,45,48,49,50,51,52] reported the percentage of their sample that engaged in active commuting, and it ranged from 12.00% to 70.41%.

#### 3.2.1. Measurement of Active Commuting

Out of all the studies, only one study measured ACS objectively, through the use of an accelerometer, specifically, an ActivPAL3 accelerometer [47]. In this study, participants had an accelerometer taped to their right thigh, and ACS in the morning was estimated with the average steps across three valid school days, multiplied by five (weekdays) and two, to account for the round trip. All other studies used self-report methods. Out of these, two studies asked parents of the participants to report their children’s mode and duration of commuting to and from school [43,52]. The rest of the studies had participants self-report their own commuting activity.

Overall, there seemed to be a lack of consensus whether ACS would constitute one-way or round-trip active commuting. Approximately half of the studies (seven) asked about engaging in active commuting only while going to the school [42,43,45,47,48,52,53], in which one of the studies doubled the duration reported, to account for the round trip [47]. The remaining five studies measured active commuting both to and from school [44,46,49,50,51]. Out of 12 studies, only six studies tried to assess the dose of ACS, albeit only in terms of duration [43,45,47,50,52,53], whereas the other studies measured it as a binary variable. There was variation in the way duration was categorized, with three studies categorizing it as <15 min or >15 min [45,52,53], one study categorizing it as <30 min, 30–60 min, and >60 min [50], one study using cut off points as <25 min, 25–50 min and >50 min [43], while one measured it as a continuous variable [45]. Apart from this, only 3 studies assessed ACS within a particular time period (e.g., ACS in the past week) [47,48,49]. Two studies used validated questions, in which one study used two items from a 7-item validated questionnaire about school travel [52], and the other study validated their questions using data from actigraphy [44]. Two more studies conducted a test-retest reliability for the questionnaire they used [46,48]. None of the studies assessed intensity of ACS. Only 4 studies assessed other types of physical activity that participants may have engaged in, and these included asking participants about their engagement in activities such as sports, extra-curricular physical activities, and any other organized exercise apart from sports, recess activity, etc.

#### 3.2.2. Measurement of Cognition and Academic Achievement

Two studies measured both cognition and academic achievement [43,48]. Including the two studies, a total of eight studies tested the association between active commute and cognition [42,43,45,47,48,49,52,53], and six studies [44,46,47,48,50,51] examined the association between active commute and academic achievement. Cognitive outcomes included domains of intelligence [45,52], executive functioning [47,48], information processing speed [47], working memory [43], verbal fluency [53], attention [43], and visuospatial skill [42,49]. The measures used were either computerized objective tasks (e.g., Eriksen flanker task), or paper-and-pencil tasks scored by the researchers (e.g., cognitive maps). The studies measuring academic achievement covered domains of reading fluency and reading comprehension [44], arithmetic skills [44,46,47,48,50,51], language scores (including English, Norwegian, and Spanish) [46,47,50,51], final subject grades [51], and grade point average [51]. Four studies used grades calculated by the school [46,47,50,51], one study used scores on a custom-made test [48], and one study used scores from a standardized achievement test battery [44]. Our results are centered around studies with adolescents (aged 10–19 years, as defined by the World Health Organization). The age range of the sample in these studies was between 10 and 19 years. If a study had an age range that overlapped with adolescents, such as ages 7–12, we did not include them in the adolescent analysis because of potential confounds.

### 3.3. Active Commute and Cognition

Table 3 summarizes the results of the association between ACS and cognition and/or academic achievement. Five studies measured the association between active commute and cognition among adolescents. Overall, three studies found a positive association with at least one cognitive measure [42,45,47]. Two studies did not find any significant association with any of the cognitive variables [48,53], and one study found no associations with some cognitive variables but not others [42].

The two studies that did not find an association with any of the cognitive variables measured executive functioning using the Eriksen flanker task [48], and verbal fluency [53]. In one of the studies, active commute included using inline skates and scooters [53], while the other included only walking and bicycling. One study showed that ACS was not associated with some cognitive variables. In this study, visuospatial skill was measured through the cognitive maps task. They found a lack of relationship richness scores [42] and explained that this result is not surprising because the task’s instructions did not emphasize the drawing of details such as landmarks, but merely chalking out the path from home to school.

Among the three studies finding positive associations, one study [42] found positive association with accuracy score on the visuospatial task, across the entire sample. The other two studies found significant associations with a subset of the sample. Girls who engaged in ACS had better overall cognitive performance, as well as improvements in numeric, verbal and reasoning subscales of intelligence as compared to girls who commuted passively [45], and girls who engaged in ACS had better performance on the d2 Test of Attention as compared to boys who engaged in ACS [47].

#### 3.3.1. Sex Difference

Two studies found a sex difference, with ACS showing an effect among girls as compared to boys. Among girls only, ACS was positively associated with verbal, numeric, and reasoning task performance, as well as overall cognitive performance [45], and performance on the d2 Test of Attention [47].

#### 3.3.2. Dose–Response Relationship

The duration of ACS was tested as a predictor of cognitive outcomes in one study. They categorized the duration as either less than 15 min or greater than 15 min and found that girls (but not boys) active commuting for greater than 15 min performed significantly better on numeric tasks, reasoning tasks, and overall cognitive performance, as compared to girls who active commuted for less than 15 min [45]. In another study, the frequency of ACS was a predictor, with cognitive map accuracy scores being better among children walking to school for more than four days of the week [42].

#### 3.3.3. Types of Cognitive Outcomes

Various types of cognitive outcomes were measured across the five studies. Broadly, they could be classified into domains of intelligence and executive functioning. Sub-domains of executive functioning included response inhibition, verbal fluency, attention, working memory and visuospatial skill, whereas sub-domains of intelligence measured were verbal, non-verbal, numeric, and reasoning abilities. The sensitivity of cognitive outcomes to physical activity is an important consideration in determining the potential effects of physical activity. Amongst the outcomes measured, prior research has shown that intelligence may not be sensitive enough to respond to changes in physical activity, whereas executive functioning may be more responsive to physical activity [54].

However, the study assessing intelligence found a significant positive association of verbal, numeric and reasoning abilities with ACS only among girls [45]. Among the studies measuring aspects of executive functioning, it was observed that ACS was associated with d2 Test of Attention [47] and accuracy scores on a task of visuospatial skill [42] but not the flanker task [48] and verbal fluency [53]. An important difference is ACS was measured objectively through an accelerometer in one study [47], thus resulting in a more accurate estimate of physical activity, as compared to self-report by students in the other studies. The other two studies that did not find significant associations did not measure ACS objectively, nor did they measure the dose of ACS. Thus, the study quality may have played a role in the significance of the findings. One of the two studies found improvement in the d2 Test of Attention only among girls, but the study assessing performance on flanker did not conduct analyses separately by sex, which may be reason for the non-significant findings.

ACS was not significantly associated with information processing speed. In both these studies, ACS was characterized as a binary variable, and the duration of ACS was not measured. Not assessing dose–response relationship may have contributed to the non-significant results.

The relationship between ACS and cognition was also explored in studies with younger children [43,49,52]. The age range in these studies was 4–12 years. Two of these studies found a positive association, across the entire sample, with performance on the n-back task [43] and aggregated spatial orientation scores [49]. One study with children aged 4–7 years old did not find an association between ACS and scores on the Differential Aptitude Test for children [52].

### 3.4. Active Commute and Academic Achievement

Five studies measured the association between active commute and academic achievement among adolescents. Overall, three studies found a significant positive association with at least one aspect of academic achievement [46,48,50], and two studies found no association with at least one academic achievement measure [47,51]. No negative associations were found among adolescents. All five studies measured arithmetic skills, with four studies measuring scores on a language test [46,47,50,51].

Among the studies finding no association, one study assessing language and arithmetic scores found no associations across the entire sample of children from grades 7–9 [47]. Similarly, the other study did not find significant associations among secondary school participants for language, mathematics, and science.

Of the three studies finding positive associations, two studies found that adolescents engaging in ACS [46] or those engaging in 30–60 min of ACS [50] had higher odds of having better academic achievement scores than those engaging in passive commuting. Average grade across subjects was used as a measure of academic achievement in both these studies. One study found that bicycling to school had a positive association with scores on a custom-made arithmetic test, but the same was not observed for walking to school [48].

#### 3.4.1. Sex Difference

Overall, two studies assessed sex difference [46,47]. Both the other studies did not observe any sex differences, with the results being similar across girls and boys.

#### 3.4.2. Dose–Response Relationship

Only one study explored a dose–response relationship, by measuring the duration of ACS. It was found that those engaging in 30–60 min had better grades in language and mathematics as compared to passive commuters [50].

The relationship between ACS and academic achievement was also explored among children with a mean age of 7 years [44] or between 6 and 12 years [51]. One study measured reading fluency and comprehension using a standardized achievement test battery, and found improved performance at baseline and after two years among those active commuting to school for greater than 14 min per day, across the sample [44]. Both studies found negative associations as well, such that ACS was associated with poorer grades, and lower odds of having high grades in all of the courses, as compare to passive commuters [51], and with reading comprehension, among girls, but not boys, who actively commuted to school [44].

### 3.5. Quantitative Analysis

**Figure 2:**
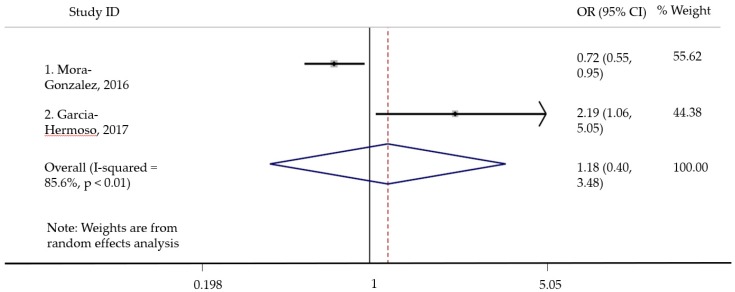
Quantitative analysis of active commuting and academic achievement.

Figure 2 summarizes the results of ACS with math performance. Math performance was used as a dichotomous (high vs. low) outcome in the studies. For one study [50] that reported odds ratio for obtaining a math performance score above level 5, 7, and 9, a combined odds ratio was calculated to be used for the analysis. In another study [51], odds ratio was calculated separately for primary and secondary school children. Only the data for secondary school children was included in the analysis, and a combined score of primary and secondary school children was not calculated. This is because age plays an important role in cognitive development of children, with structural and functional brain changes being different across pre-adolescent and adolescent youth [55]. The data for secondary school children was included over primary school children because it matched the sample age range of the other study included in the analysis, and because our results were focused on adolescents. The results showed that engaging in ACS was not significantly associated with math performance. Compared to those engaging in passive commuting, engaging in ACS was not associated with math performance (pooled odds ratio = 1.18; CI = 0.40, 3.48; p = 0.77). Owing to the small sample size for the analysis, publication bias tests were not performed.

Two studies measured math performance as a continuous variable. However, a quantitative analysis could not be performed due to lack of match between outcome measures. Two studies had the same outcome assessment for visuospatial skill, and two studies had the same outcome assessment for academic achievement. However, a quantitative analysis could not be performed in either of these cases because of lack of match between the statistical techniques.

### 3.6. Study Quality Assessment

Table 4 shows the study quality assessment scores for each study. Out of a maximum score of 14, the average score across the 12 studies was 7.50. Among these, the average score for studies with adolescents was 7.13. Research questions and objectives were all (100%) described clearly, and all but one study defined the study population clearly. Most studies controlled for some covariates or confounding factors during analysis, but only four studies [44,45,47,50] controlled for other types of physical activity participants may have engaged in. Five studies controlled for socioeconomic status [43,48,49,50,52] and six studies controlled for parental education [44,45,46,47,49,52] as a proxy for socioeconomic status. Overall, eight studies accounted for socioeconomic status by one of the two methods. Eight studies accounted for age [43,44,45,47,48,49,51,52], either using their chronological age, or their school grade level (two studies). Among the potential covariates of physical activity, sex, age, body mass index, and socioeconomic status and/or parental education, studies that controlled for 2 or more covariates were given a score of 1. All studies used clearly defined, valid and reliable outcome measures. The same was not true of the exposure measures (engagement in ACS), as most studies used self-reported measures that were not validated. Except two studies, the participation rate of eligible participants was greater than 50.00%, ranging between 60.00% and 99.13% and with an average participation rate of 77.71% (SD = 13.80%). This was determined from the recruitment statistics reported by the studies. Participation rate refers to the percentage of participants who participated in the study from the pool of eligible participants that were approached for the study. Only one study provided a sample justification using a power analysis, and some other studies provided effect size estimates. With the exception of the two prospective studies, the others did not measure exposure before the outcome, and none measured exposure more than once. All study descriptions omitted any mention about potential blinding of the assessors, resulting in a score of 0 (not determined) for all the studies on that criterion. Approximately half of the studies measured different levels of exposure, and they defined ACS via an ordered categorical or continuous scale and the remainder quantified ACS as a dichotomous (yes/no) variable. The sample of one of the studies was from a larger study, and they did not explain the recruitment methods in detail [42]. However, this study did not cite the original article, and we were unable to determine the details of the recruitment process. Excluding this study from the study quality assessment did not change the average study quality score much (7.50 vs. 7.27). Only four studies scored above the median value of 7. All of them largely showed positive associations between ACS and cognition or academic achievement.

## 4. Discussion

This article presents a systematic review of 12 studies examining the relationship of ACS with cognitive performance and academic achievement, among children and adolescents. This is a new area of research, with the earliest study being as recent as 2011. These studies were conducted across nine countries, with none of the studies being conducted in the US. There were more studies conducted in Spain (n = 4) than any other country (n = 1 each). The median sample size was 568, and participants’ age ranged from 4 to 18.5 years. For cognition, three among five studies found a positive relationship between ACS and at least one cognitive measure, including two studies that found positive associations among girls, but not boys. One study found a dose-relationship with cognition, such that those engaging in a higher duration of ACS showed a significant relationship as compared to those engaging in a lower duration. Three of the five studies found a positive relationship between ACS and at least one measure of academic achievement; one study found a dose–response relationship, and no sex differences were found. We conducted a quantitative analysis to estimate the effect of the association between ACS and mathematics performance, and found a small, non-significant positive association with math performance for children who engaged in ACS. Among children, similar results were observed, with two of three studies finding a positive association with cognition, and one of two finding a positive association with academic achievement.

Generally, the evidence for active commuting relationships with cognition and academic achievement was in the positive direction, with three of five studies (for cognition and academic achievement, each) finding significant positive results, among adolescents. Similarly, five out of eight (for cognition) and four out of six (for academic achievement) found significant positive results, across children and adolescents. Despite this, definite conclusions cannot be made. Two studies with adolescent samples found that ACS was associated with cognitive performance only among girls. The authors [47] theorized that this might be due to a higher level of stress experienced by adolescent girls as compared to boys [56]. Thus, girls may benefit more than boys from the stress reduction effects of physical activity, by buffering the established detrimental effects of stress on cognitive performance [57]. They also theorized that hormonal differences between adolescent girls and boys may play a role. Estrogen among females interacts with insulin growth factor-I to improve neuronal survival [58]. An increase in exercise is associated with an increase in insulin growth factor levels [59], and hence girls may gain an advantage during this developmental phase. However, different hormones have been shown to have different neuroanatomical effects that would support potential cognitive development among girls *and* boys [60]. Additionally, physical activity may reduce anxiety and depressive symptoms among girls thus improving cognition [45], but one study found that depressive symptoms did not mediate the relationship between ACS and cognition [47].

ACS was positively associated with visuospatial skill, working memory, attention, and general intelligence, as well as with grades, arithmetic scores, and reading fluency and comprehension. These results were similar to those found with LTPA, which is more widely studied among this age group. Prior research with LTPA has shown that it is associated with improvements in cognition and academic achievement [3]. ACS was not associated with executive functioning, general intelligence, aspects of visuospatial skill, as well as language and arithmetic scores. It was negatively associated with some aspects of visuospatial skill, grades, and reading comprehension (among girls). While these results contradict those found with respect to LTPA, it is possible that methodological issues as well as key factors associated with ACS may account for these findings, as elaborated below.

Overall, the evidence may be considered weak due to the methodological issues pertaining to the original studies. A majority of the studies (n = 10) were cross-sectional studies, and only two studies were prospective in nature. Cross sectional studies preclude a causal interpretation of the relationships and provide a snapshot of the behavior at only one point in time. The majority of the studies did not rely on objective measurement methods for ACS. Whereas cognition and academic achievement were measured objectively, all except one study (n = 11) used self-report questionnaires to measure ACS. Retrospective self-report increases measurement error due to misreporting, as children/adolescents may not accurately estimate or remember their physical activity and may be susceptible to social desirability bias [61,62,63]. That is, children/adolescents may over-report their ACS levels in order to present themselves as being physically active, which is a socially desirable behavior [61]. Furthermore, the self-report instruments used to assess ACS lacked scientific rigor. While most of the self-report questions did not use a validated self-report measure, they also did not establish validity or reliability of the self-report measures used, with the exception of two studies that tested validity and two that tested reliability.

Additionally, more than half of the studies did not measure the duration and/or intensity of ACS. Of the two studies that measured duration, a dose–response relationship was found, such that higher duration was associated with positive benefits, as compared to lower duration of ACS. This is consistent with previous studies that have observed similar dose–response relationships with cognition [64]. For example, in an after-school LTPA program, it was found that those engaging in 40 min of physical activity five days/week had greater executive function and mathematics performance as compared to those who were engaging in only 20 min of physical activity for the same number of days [5]. In another study, those attending more after-school physical activity sessions had larger improvement in executive functioning [7]. Considering this evidence regarding the dose–response relationship of physical activity, inadequate estimates of active commuting engagement may underestimate the relationship and preclude an in-depth understanding of its association to cognition and academic achievement. Similar results are found among older adults engaging in active commuting, such as those engaging in >60 min of active commuting may have a lower risk of dementia [65]. In another study, active commute along with a good built environment was associated with better cognitive functioning [66]. Apart from this, only four studies controlled for the other types of physical activity engagement. Given the literature between LTPA and cognition discussed above, it is important for future studies to account for other physical activity participation.

Along with methodological issues, key factors related to ACS must be taken into consideration while interpreting the results. For example, participants may have engaged in ACS alone or with a group, and group activity can influence enjoyment and activity type, intensity, duration, etc. [67], thereby increasing the dose received and its impact on cognition. Apart from this, because ACS involves walking or cycling to school, it would involve exposure to automobile traffic and air pollutants. Research has shown that air pollution is associated with poor cognitive and academic performance in children [68], as well as slower brain maturation [69]. For example, exposure to air pollution measured by isophorone in the ambient air was associated with a significantly lower mathematics score by 1.63 points in early childhood [70]. These negative effects have been shown to start around kindergarten and continue until elementary school [71]. In a longitudinal study, exposure to air pollutants during ACS was associated with a reduction in the growth of working memory over the course of a year [72]. In this review, only one of the studies controlled for environmental pollutants and neighborhood features [43], and thus, the results for other studies should be interpreted keeping this issue in mind. In line with this, it is also important to consider the reason for active vs. passive commute to school and the implications it has for cognitive and academic development. Children who may be active commuting for long durations because of non-availability of other resources, may not show beneficial cognitive effects possibly because of having to active commute in spite of air quality and environmental factors. However, children that purposefully choose to engage in ACS to improve their health may show beneficial effects, especially if they choose active commuting after considering the air quality and other environmental factors. While half of the studies controlled for socioeconomic status and parental education, none of the studies controlled for reasons for engaging in ACS, and how that may mediate the effect on their outcomes. Safety is another key factor as parental figures and guardians are key gatekeepers of their child’s ACS. Studies have shown that children’s engagement in ACS is influenced by parents’ perceptions of neighborhood safety and crime [73,74] as well as distance [75]. Apart from this, the built environment, such as access to safe sidewalks and traffic lights on the school route can affect engagement in ACS. For example, children from high income neighborhoods engaged in significantly less active commuting if they lived in low-walkable as compared to high walkable neighborhoods [73]. Similarly, children passing through safe school routes featuring sidewalks, traffic lights, safe pedestrian crossing and bicycle paths, were more likely to engage in ACS than those who were not passing these routes while going to school [76]. Other factors such as home–school distance, automobile traffic, and living in rural or urban neighborhood [77] may affect engagement in ACS and the potential cognitive benefit derived from it. However, we do not have a good understanding of the interaction of these factors with ACS and its impact on cognitive functioning.

The quantitative analysis result showing that ACS was not statistically related to mathematics contradicts the literature regarding the most commonly studied physical activity, i.e., LTPA and academic achievement. ACS is different from LTPA. The former involves engaging merely in walking and/or bicycling for the purpose of getting from one place to another, whereas LTPA can include a variety of physical activities, such as playing with friends at the playground, engaging in sports or dance, or doing after-school physical activity programs. A number of studies have shown small [2] or large [3] positive effects of LTPA with academic achievement in terms of mathematics performance [2] in this population. It may be that LTPA yields itself to greater cognitive benefits as it calls for more neuromuscular complexity as compared to walking or bicycling [78]. However, the results could very well be due to the shortcomings of this analysis and therefore, must be interpreted very cautiously. This analysis is not representative of the literature on this topic, as it involved only two out of the six possible studies. We could not include some studies due to a lack of homogeneity in measures, or effect size reporting, as a result of which this analysis is not an accurate estimation of the effect size. One study did not account for LTPA, in the analysis, which has the potential to explain some of the findings [51]. Because LTPA has a positive relationship with academic achievement, it is important to account for the amount of LTPA that the two groups (active commuting vs. not) may be engaging in. Other methodological issues pertaining to the original studies discussed in the section before should be accounted for while interpreting the results.

The strength and limitations of this review should be considered when interpreting the findings. This is the first study to systematically review literature on ACS and cognition or academic achievement and provide recommendations for future research. One of the main limitations is that we included studies only published in the English language, and some studies published in regional language may have been excluded. The ACS in this review mostly consisted of walking and/or bicycling. There are different modes of transport such as electric bikes, skates and scooters that can be argued as being a part of active commuting and may or may not impact cognition. Only one study in our review included explicit measurement of these tandem activities as forms of active commuting [53]. Therefore, use of these transportation devices cannot be teased apart and could confound our findings. Additionally, none of the studies reviewed had a control group. Lack of control trials regarding ACS and its association with cognition and/or academic achievement make it difficult to separate the influence of maturation during childhood and adolescence. Without a randomized control trial, we cannot determine if children and adolescents would have experienced any of these positive cognitive effects naturally. We used broad inclusionary criteria for this study, given the lack of research in this field, with the purpose of providing guidance for future interventions. Accordingly, our conclusions are broad, and based on outcomes and exposures that are not identical to each other.

Future studies need to implement more rigorous, longitudinal designs (e.g., randomized controlled trials), using objective assessments of physical activity (e.g., using accelerometers validated for use with research), to assess the strength of the relationship between ACS and cognition or academic achievement. If self-report measures are used to assess ACS, the validity and reliability of these measures should be established, and researchers should consider multiple assessments over the course of an academic year to acquire a robust measure of ACS engagement. Such a measure would differentiate modality, frequency, intensity, duration, and involvement with others (group vs. individual activity) to determine dose–response relationships with other outcomes of interest. Moreover, researchers should ensure all assessments are age, environmentally and culturally appropriate. Such methods will enable a detailed understanding of active commute and its relationships with changes in cognition and academic achievement.

## 5. Conclusions

This article is a systematic review of the relationship of ACS with cognitive performance and academic achievement. Our findings reveal that ACS has the potential to influence cognition and academic achievement, but clear conclusions cannot be drawn based on the current state of evidence. Future studies with longitudinal methodological designs are needed to further understand this relationship.

## Figures and Tables

**Table 1:** Basic characteristics of studies included in the systematic review.

ID	First Author (Year)	Country	Sample Size	Sample Characteristics	Age/Grade Range	Mean Age in Years (SD)	Female %	% Engaging in Active Commute	Study Design	Statistical Model
1	Martínez-Gómez (2011)	Spain	1700	Adolescents in the AVENA study	Ages 13–18.5	15.4 (1.3)	52.47	Boys = 64% Girls = 67%	Cross sectional	Analysis of covariance
2	Haapala (2014)	Finland	186	Children from the Physical Activity and Nutrition in Children study and the First Steps Study	Grades 1–3	7.7 (0.4)	42.47	-	Prospective	Analysis of covariance
3	Stea (2014)	Norway	2432	Part of the “Active and Healthy Youth” intervention study	Grades 7–9	16 (0.4)	51.72	-	Cross sectional	Multiple logistic regression
4	Van Dijk (2014)	Netherlands	270	Part of the GOALS study	Grades 7–9	13.4 (1.31)	47	-	Cross sectional	Multiple linear regression
5	Domazet (2016)	Denmark	568	Baseline data from the LCoMotion-Learning, Cognition and Motion study.	Grades 6–7	Boys = 13 (0.6), girls = 12.9 (0.6)	52.64	Boys = 37% Girls = 36.4%	Cross sectional	Mixed Model Regression
6	López-Vicente (2016)	Spain	2897	Part of the BRain dEvelopment and Air polluTion ultrafine particles in scHool children (BREATHE) project.	Grades 2–4	8.6 (0.9)	49.7	1–25 min: 18%; 25–50 min: 21%; > than 50 min: 12%	Cross sectional and Prospective	Linear Mixed Effects Model
7	Fang (2017)	Taiwan	521	-	Grades 1–6	Mean grade level = 3.62 (0.46)	50.1	49%	Cross sectional	Ordinal Least Squares method
8	García-Hermoso (2017)	Chile	389	-	Grade 7	12 (0.6)	48.3	23%	Cross sectional	Analysis of Covariance
9	Mora-Gonzalez (2017)	Spain	2138	-	1. Grades 1–6 2. Grades 7–10	1. 9.96 (1.23) 2. (14.24 (1.26)	1. 50.9 2. 50.3	Primary school: Boys = 70.4% Girls = 62.2%. Secondary school: Boys = 65% Girls = 65.9%	Cross sectional	Analysis of Covariance
10	Moran (2017)	Israel	92	-	Grades 5–6	Not provided	53.3	-	Cross sectional	Multivariate linear regression
11	Westman (2017)	Sweden	345	-	Grades 4–8	Not provided	47.8	-	Cross sectional	Analysis of Variance
12	Ruiz-Hermosa (2018)	Spain	1159	Baseline data from the MOVI_KIDS intervention	Ages 4–7	5.3 (0.6)	48.31	46%	Cross sectional	Analysis of Covariance

**Table 2:** Measurement of active commuting, cognition and academic achievement.

ID	Type of Active Commute	Measure of Active Commute	Active Commute Measurement	Cognitive Domain	Cognitive Measure	Academic Achievement Domain	Academic Achievement Measure
1	Walking and/or cycling	Self-report	1. ‘How do you usually travel to school?’ 2. ‘How long does it usually take you to travel from home to school?’	Intelligence (verbal, numeric and reasoning abilities)	Spanish version of the SRA Test of Educational Ability	-	-
2	Walking and/or cycling	Self-report	PANIC Physical Activity Questionnaire	-	-	1. Reading fluency 2. Reading comprehension 3. Arithmetic skills	1. Subtest of the Reading Achievement Test battery (ALLU battery) 2. Subtest from the ALLU battery 3. Basic arithmetic test
3	Walking and/or cycling	Self-report	‘How do you usually commute to/from school?’	-	-	Language and arithmetic	Grades from Norwegian, English and Mathematics courses
4	Walking and/or cycling	Accelerometry	ActivPAL3 accelerometer data from 3 valid weekdays	1. Executive functioning (Response inhibition and selective attention) 2. Information processing speed	1. d2 Test of Attention 2. Symbol Digit Modalities Test	Language and arithmetic	Grades from Norwegian, English and Mathematics courses
5	1. Walking 2. Cycling	Self-report	Participants were asked how they arrived to school	Executive function (Inhibitory control)	Eriksen flanker task	Arithmetic	Score on a custom-made Mathematics test
6	Not mentioned	Self-report	Questionnaire asking parents to report their children’s mode and duration of transport	1. Working Memory 2. Attention	1. N-back task 2. Attentional Network Task	-	-
7	Walking and/or cycling	Self-report	Questionnaire asking about mode, distance, time and number of stops while traveling to school	Visuospatial skill	Cognitive map of the home–school route	-	-
8	Walking	Self-report	1. ‘How do you usually travel from home to school and from school to home?’ 2. ‘How long does it usually take you to travel from home to school and from school to home?’	-	-	Language and arithmetic	Grades in Mathematics and language courses
9	Walking and/or cycling	Self-report	Two questions regarding how participants travelled to school and traveled back from school			Language and arithmetic	1. Final grades at the end of the academic year for English, Spanish, Mathematics natural sciences, and social sciences courses 2. Grade Point Average
10	Walking and/or cycling	Self-report	Brief survey regarding school travel mode	Visuospatial skill	Sketch map of the home–school route	-	-
11	Walking and/or cycling	Self-report	Research staff asked students their travel mode and duration	Verbal fluency	Word fluency task	-	-
12	Walking and/or cycling	Self-report by parents	Parents were asked 2 questions, taken from a 7-item school travel survey: 1. ‘How does your son/daughter usually go from home to school?’ 2. ‘How long does it take for your son/daughter to go from home to school?’	1. Verbal and non-verbal intelligence, also summarized as general intelligence 2. Logical reasoning, verbal factor, numerical factor, and spatial factor, also summarized as general intelligence	1. Battery of General and Differential Aptitudes for children aged 3–6 2. Battery of General and Differential Aptitudes for children aged 6–8 years old.	-	-

*Note*. AVENA = Alimentación y Valoración del Estado Nutricional de los Adolescentes [Feeding and assessment of nutritional status of Spanish adolescents]; GOALS = Grootschalig Onderzoek naar Activiteiten van Limburgse Scholieren [Large-scale Research of Activities of Limburgs Students]; MOVI_KIDS = Multidimensional physical activity intervention during two years in pre-school children; SRA = Science Research Associate; PANIC = Physical Activity and Nutrition In Children study; ALLU = Ala-asteen lukutesti [Reading test for primary school].

**Table 3:** Results of active commuting to school (ACS), cognition, and academic achievement.

ID	Estimated Relationship between Active Commute and Cognition	Estimated Relationship between Active Commute and Academic Achievement	Qualitative Brief Summary for Cognition	Qualitative Brief Summary for Academic Achievement
1	Girls in the group with ACS longer than 15 min had significantly higher scores in verbal ability (score +2.75; 95% CI, 1.18–4.32), numeric ability (score +1.94; 95% CI, 0.71–3.17), reasoning ability (score +2.19; 95% CI, 0.81–3.57), and overall cognitive performance (score +7.06; 95% CI, 3.57–10.55) than girls in the non-ACS group (all *p* < 0.01); no statistically significant association among boys.	-	Among girls but not boys, active commute significantly associated with better verbal, numeric, reasoning and overall cognitive performance.	-
2	-	Children in the upper half of physically active school transportation in Grade 1 (≥median of 14 min/day) had a better reading fluency in Grades 1–3 than those who were in the lower half after adjusting for age, sex, parental education and the PANIC study group (*p* = 0.038, η^2p^ = 0.02). Boys active commuting in Grade 1 (≥median of 14 min/d) had a better reading fluency and reading comprehension in Grades 1–3. Among girls, active commuting in grade 1 was inversely associated with reading comprehension in Grade 3.	-	Among all children, those active commuting for >14 min in grade 1 had significantly better reading fluency scores in grades 1 and 3.
3	-	High academic achievement was associated with active commuting to school among girls (AOR: 1.51 (1.10, 2.08)) and boys (AOR: 1.72 (1.26, 2.35)).	-	Active commuting was associated with high academic achievement in both boys and girls.
4	Active commuting to school was not significantly associated with performance on the d2 Test of Attention (*β* = 0.05) and the Symbol Digit Modalities Test (*β* = 0.04). Simple slopes analyses revealed a significantly positive association between active commuting to school and performance on the d2 Test of Attention in girls (*β*= 0.17, *p* = 0.04), but no significant association in boys (*β* = −0.03, *p* = 0.66).	Active commuting to school was not significantly associated with academic achievement (*β* = 0.04) and mathematics achievement (*β* = 0.08).	Active commuting was significantly associated with executive function among girls only, and not associated with information processing speed among both boys and girls.	Active commuting was not significantly associated with academic or math achievement.
5	Active commuting to school, in terms of walking or bicycling was not significantly associated with interference scores on reaction time (walking: *β* = −1.4 (−6.9, 9.8), *p* = 0.74; cycling: *β* = 4.0 (−2.7, 10.6), *p* = 0.24) or accuracy (walking: *β* = −0.5 (−2.7, 1.6), *p* = 0.64; cycling: *β* = −0.1 (−1.8, 1.7), *p* = 0.93) on the Eriksen flanker task.	Bicycling to school was associated with superior mathematics performance as compared to passive transportation (*β* = 5.4 (1.9, 8.8), *p* < 0.01), and walking to school was not associated with mathematics performance (*β* = 0.1 (−4.1, 4.4), *p* = 0.95).	Active commuting was not significantly associated with executive functioning.	Cycling to school was associated with better mathematics performance as compared to using passive commuting.
6	More than 50 min of active commuting to school was associated with 9.9 d’ point greater 3-back baseline score and their 2-back growth was 11.2 d’ points below passive commuters.	-	More than 50 min of active commuting was associated with better performance on 3 back at baseline and lower performance on 2 back at 1 year	-
7	Active commuting to school was positively related to the number of objects, correctness of route orientation and aggregated scores, and negatively associated with correctness of route structure for the spatial cognition maps of the participants (all *p* < 0.01). Active commuting was not associated with the number of landmarks, paths and places in the participants’ cognitive maps.	-	Active travel was positively associated with 3 aspects of the cognitive maps, negatively associated with route structure correctness, and not associated with 3 aspects of the maps.	-
8	-	Children with 30 to 60 min of active commuting to school were more likely to have a better academic achievement than non-commuters (language, OR = 3.53, (1.12, 4.37); *p* < 0.01; mathematics, OR = 2.19 (1.06, 5.05); *p* = 0.03). There were no statistically significant differences between those passive commuting, active commuting for < 30 min, or active commuting for > 60 min.	-	Engaging in 30-60 min of active commuting was significantly associated with better grades in language and mathematics, as compared to passive commuting.
9	-	Passive primary school commuters had better grades in math (7.46 ± 0.17 vs. 6.95 ± 0.12, *p* < 0.01), Spanish (7.72 ± 0.16 vs. 7.10 ± 0.12, *p* < 0.01), English (7.63 ± 0.17 vs. 7.01 ± 0.12, *p* < 0.01), natural sciences (7.59 ± 0.17 vs. 7.02 ± 0.12, *p* < 0.01) and grade point average (7.60 ± 0.15 vs. 7.02 ± 0.11, *p* = 0.01) than active commuters, whereas no significant associations were found in the secondary school students for all the selected subjects and the grade point average (all *p* ≥ 0.06). Active primary school children had lower odds of having high grades for math (OR = 0.65 (0.43, 0.98)), Spanish (OR = 0.57 (0.38, 0.86)), English (OR = 0.48; (0.32, 0.73)) and grade point average (OR = 0.64; (0.41, 1.00)). There were no statistically significant associations for secondary school children.	-	Among primary school children, engaging in active commuting was associated with poorer grade point average and lower grades in Mathematics, Spanish, English and natural sciences, as compared to passive commuters. No significant associations were found among secondary school children.
10	The accuracy scores obtained from maps of children who walk to school most of the week (at least four out of six school-days) were significantly higher than those of children who did not (M = 8.69 vs. M = 7.71, *t* (90) = −3.66, *p* < 0.01). The richness scores of the sketch maps did not differ according to the children’s school travel mode.	-	Active commuters had significantly better accuracy scores but not with the richness scores on the cognitive maps, as compared to passive commuters.	-
11	A 3 (grade) by 2 (sex) by 3 (travel mode) ANOVA only yielded main effects of grade, *F*(2290) = 34.20, *p* < 0.01 and sex, *F*(1290) = 35.45, *p* < 0.01.		Active commuting was not associated with scores on the word fluency task	
12	Walking to school (vs. passive commuting) was not significantly associated with general verbal intelligence (38.50 ± 7.76 vs. 40 ± 6.81, *p* = 0.54 and non-verbal intelligence (37.80 ± 8.25 vs. 39 ± 7.61, *p* = 0.97) and general intelligence (76.4 ± 14.94 vs. 79.10 ± 13.18, *p* = 0.76) among preschoolers. Among primary school children, walking was not significantly associated with logical reasoning (27.60 ± 9.50 vs. 28.00 ± 8.37, *p* = 0.80), verbal factor (21.80 ± 6.37 vs. 22.40 ± 5.27, *p* = 0.76), numerical factor (16.40 ± 8 vs. 17.40 ± 7.89, *p* = 0.47), spatial factor (15.90 ± 7.11 vs. 16.00 ± 6.30, *p* = 0.64) and general intelligence (54.20 ± 18.10 vs. 55.80 ± 15.89, *p* = 0.80).	-	Active commuting and its duration were not significantly associated with cognitive performance	-

**Table 4:** Study quality assessment.

Criteria	1	2	3	4	5	6	7	8	9	10	11	12
1. Was the research question or objective in this paper clearly stated?	1	1	1	1	1	1	1	1	1	1	1	1
2. Was the study population clearly specified and defined?	1	1	0	1	1	1	1	1	1	1	1	1
3. Was the participation rate of eligible persons at least 50%?	1	1	1	1	1	1	1	1	0	0	1	1
4. Were all the subjects selected or recruited from the same or similar populations (including the same time period)? Were inclusion and exclusion criteria for being in the study prespecified and applied uniformly to all participants?	1	0	0	0	0	0	0	1	0	0	0	0
5. Was a sample size justification, power description, or variance and effect estimates provided?	0	1	1	1	1	1	0	1	1	1	0	0
6. For the analyses in this paper, were the exposure(s) of interest measured prior to the outcome(s) being measured?	0	1	0	0	0	1	0	0	0	0	0	0
7. Was the timeframe sufficient so that one could reasonably expect to see an association between exposure and outcome if it existed?	0	1	0	0	0	1	0	0	0	0	0	0
8. For exposures that can vary in amount or level, did the study examine different levels of the exposure as related to the outcome (e.g., categories of exposure, or exposure measured as continuous variable)?	0	1	0	1	0	1	0	1	0	1	1	1
9. Were the exposure measures (independent variables) clearly defined, valid, reliable, and implemented consistently across all study participants?	0	0	0	1	0	0	0	0	0	0	0	0
10. Was the exposure(s) assessed more than once over time?	0	0	0	0	0	0	0	0	0	0	0	0
11. Were the outcome measures (dependent variables) clearly defined, valid, reliable, and implemented consistently across all study participants?	1	1	1	1	1	1	1	1	1	1	1	1
12. Were the outcome assessors blinded to the exposure status of participants?	0	0	0	0	0	0	0	0	0	0	0	0
13. Was loss to follow-up after baseline 20% or less?	1	1	1	1	1	1	1	1	1	1	1	1
14. Were key potential confounding variables measured and adjusted statistically for their impact on the relationship between exposure(s) and outcome(s)?	1	1	1	1	1	1	1	1	1	0	1	1
Total Score	7	10	6	9	7	10	6	9	6	6	7	7

## Appendix A

**Table A1:** PRISMA Checklist.

Section/Topic	#	Checklist Item	Reported on Page #
**TITLE**			
Title	1	Identify the report as a systematic review, meta-analysis, or both.	Manuscript title
**ABSTRACT**			
Structured summary	2	Provide a structured summary including, as applicable: background; objectives; data sources; study eligibility criteria, participants, and interventions; study appraisal and synthesis methods; results; limitations; conclusions and implications of key findings; systematic review registration number.	Abstract section
**INTRODUCTION**			
Rationale	3	Describe the rationale for the review in the context of what is already known.	2–3
Objectives	4	Provide an explicit statement of questions being addressed with reference to participants, interventions, comparisons, outcomes, and study design (PICOS).	3
**METHODS**			
Protocol and registration	5	Indicate whether a review protocol exists, if and where it can be accessed (e.g., Web address), and, if available, provide registration information including registration number.	3
Eligibility criteria	6	Specify study characteristics (e.g., PICOS, length of follow-up) and report characteristics (e.g., years considered, language, publication status) used as criteria for eligibility, giving rationale.	3
Information sources	7	Describe all information sources (e.g., databases with dates of coverage, contact with study authors to identify additional studies) in the search and date last searched.	3
Search	8	Present full electronic search strategy for at least one database, including any limits used, such that it could be repeated.	3 and Appendix B
Study selection	9	State the process for selecting studies (i.e., screening, eligibility, included in systematic review, and, if applicable, included in the meta-analysis).	3, 4
Data collection process	10	Describe method of data extraction from reports (e.g., piloted forms, independently, in duplicate) and any processes for obtaining and confirming data from investigators.	3
Data items	11	List and define all variables for which data were sought (e.g., PICOS, funding sources) and any assumptions and simplifications made.	3
Risk of bias in individual studies	12	Describe methods used for assessing the risk of bias of individual studies (including specification of whether this was done at the study or outcome level), and how this information is to be used in any data synthesis.	4
Summary measures	13	State the principal summary measures (e.g., risk ratio, difference in means).	4
Synthesis of results	14	Describe the methods of handling data and combining results of studies, if done, including measures of consistency (e.g., I2) for each meta-analysis.	4
Risk of bias across studies	15	Specify any assessment of the risk of bias that may affect the cumulative evidence (e.g., publication bias, selective reporting within studies).	4
Additional analyses	16	Describe methods of additional analyses (e.g., sensitivity or subgroup analyses, meta-regression), if done, indicating which were pre-specified.	N/A
**RESULTS**			
Study selection	17	Give numbers of studies screened, assessed for eligibility, and included in the review, with reasons for exclusions at each stage, ideally with a flow diagram.	5–6
Study characteristics	18	For each study, present characteristics for which data were extracted (e.g., study size, PICOS, follow-up period) and provide the citations.	5–6 (Table 1)
Risk of bias within studies	19	Present data on the risk of bias of each study and, if available, any outcome level assessment (see item 12).	18
Results of individual studies	20	For all outcomes considered (benefits or harms), present, for each study: (a) simple summary data for each intervention group (b) effect estimates and confidence intervals, ideally with a forest plot.	19
Synthesis of results	21	Present results of each meta-analysis done, including confidence intervals and measures of consistency.	18–19 (Figure 2)
Risk of bias across studies	22	Present results of any assessment of the risk of bias across studies (see Item 15).	18
Additional analysis	23	Give results of additional analyses, if done (e.g., sensitivity or subgroup analyses, meta-regression [see Item 16]).	N/A
**DISCUSSION**			
Summary of evidence	24	Summarize the main findings including the strength of evidence for each main outcome; consider their relevance to key groups (e.g., health care providers, users, and policy makers).	19
Limitations	25	Discuss limitations at study and outcome level (e.g., risk of bias), and at review-level (e.g., incomplete retrieval of identified research, reporting bias).	21
Conclusions	26	Provide a general interpretation of the results in the context of other evidence, and implications for future research.	21
**FUNDING**			
Funding	27	Describe sources of funding for the systematic review and other support (e.g., supply of data); role of funders for the systematic review.	N/A; not funded

## Appendix B

Search terms for each of the four databases.

Search conducted on 23 February 2019.

### Appendix B.1. PubMed

(“active travel”[tiab] OR “active commut*”[tiab] OR walk*[tiab] OR bike[tiab] OR biking [tiab] OR cycling[tiab] OR bicycling[tiab] OR bicycle[tiab] OR cycle[tiab]OR transport[tiab] OR transportation[tiab]) AND (school*[tiab] OR “schools”[MeSH]) AND (cognition[tiab] OR “cognition”[MeSH] OR “cognitive function*” [tiab] OR “cognitive performance” [tiab] OR “cognitive abilit*” [tiab] OR “executive function*” [tiab] OR “executive function”[MeSH] OR attention[tiab] OR inattentiveness[tiab] OR “working memory” [tiab] OR “information processing” [tiab] OR “response inhibition” [tiab] OR inhibition[tiab] OR memory[tiab] OR memory[MeSH] OR “verbal abilit*”[tiab] OR “numeric abilit*”[tiab] OR “reasoning abilit*”[tiab] OR “academic achievement” [tiab] OR “achievement score*”[tiab] OR “academic success”[MeSH] OR “language score*”[tiab] OR “reading score*”[tiab] OR “reading performance” [tiab] OR “math score*”[tiab] OR “math performance” [tiab] OR “mathematics score*”[tiab] OR “mathematics performance”[tiab] OR “mathematic score*”[tiab] OR “mathematic performance”[tiab] OR “academic grade*”[tiab] OR “standardized test score*”[tiab]) AND (child*[tiab] OR “child”[MeSH] OR “school children” [tiab] OR student*[tiab] OR “students”[MeSH] OR kid*[tiab] OR “school-goer*”[tiab] OR “children in school” [tiab] OR “school going children” [tiab] OR schoolchildren[tiab] OR adolescen*[tiab] OR “adolescent”[MeSH] OR teenage*[tiab] OR “teen-age*”[tiab])

### Appendix B.2. PsycInfo: Filtered by Article Type (Peer-Reviewed) and Language (English)

ti,ab(“active travel”) OR ti,ab(“active commut*”) OR ti,ab(walk*) OR ti,ab(bike) OR ti,ab(biking) OR ti,ab(cycling) OR ti,ab(bicycling) OR ti,ab(bicycle) OR ti,ab(cycle) OR ti,ab(transport) OR ti,ab(transportation) AND

ti,ab(school*) AND

ti,ab(cognition) OR ti,ab(“cognitive function*”) OR ti,ab(“cognitive performance”) OR ti,ab(“cognitive abilit*”) OR ti,ab(“executive function*”) OR ti,ab(attention) OR ti,ab(inattentiveness) OR ti,ab(“working memory”) OR ti,ab(“information processing”) OR ti,ab(“response inhibition”) OR ti,ab(inhibition) OR ti,ab(memory) OR ti,ab(“verbal abilit*”) OR ti,ab(“numeric abilit*”) OR ti,ab(“reasoning abilit*”) OR ti,ab(“academic achievement”) OR ti,ab(“achievement score*”) OR ti,ab(“academic success”) OR ti,ab(“language score*”) OR ti,ab(“reading score*”) OR ti,ab(“reading performance”) OR ti,ab(“math score*”) OR ti,ab(“math performance”) OR ti,ab(“mathematics score*”) OR ti,ab(“mathematics performance”) OR ti,ab(“mathematic score*”) OR ti,ab(“mathematic performance”) OR ti,ab(“academic grade*”) OR ti,ab(“standardized test score*”) AND

ti,ab(child*) OR ti,ab(“school children”) OR ti,ab(student*) OR ti,ab(kid*) OR ti,ab(“school-goer*”) OR ti,ab(“children in school”) OR ti,ab(“school going children”) OR ti,ab(schoolchildren) OR ti,ab(adolescen*) OR ti,ab(teenage*) OR ti,ab(“teen-age*”)

### Appendix B.3. Web of Science: Filtered by Topic, Article Type (Peer-Reviewed) and Language (English)

(“active travel” OR “active commut*” OR walk* bike OR biking OR cycling OR bicycling OR bicycle OR cycle OR transport OR transportation) AND

(school*) AND

(cognition OR “cognitive function*” OR “cognitive performance” OR “cognitive abilit*” OR “executive function*” OR attention OR inattentiveness OR “working memory” OR “information processing” OR “response inhibition” OR inhibition OR memory OR “verbal abilit*” OR “numeric abilit*” OR “reasoning abilit*” OR “academic achievement” OR “achievement score*” OR “academic success” OR “language score*” OR “reading score*” OR “reading performance” OR “math score*” OR “math performance” OR “mathematics score*” OR “mathematics performance” OR “mathematic score*” OR “mathematic performance” OR academic grade* OR standardized test score*) AND

(child* OR “school children” OR student* OR kid* OR “school-goer*” OR “children in school” OR “school going children” OR schoolchildren OR adolescen* OR teenage* OR teen-age*)

### Appendix B.4. Cochrane Library: Filtered by Title, Abstract and Keyword

(“active travel” OR active NEXT commut* OR walk* bike OR biking OR cycling OR bicycling OR bicycle OR cycle OR transport OR transportation) AND

(school*) AND

(cognition OR cognitive NEXT function* OR “cognitive performance” OR cognitive NEXT abilit* OR executive NEXT function* OR attention OR inattentiveness OR “working memory” OR “information processing” OR “response inhibition” OR inhibition OR memory OR verbal NEXT abilit* OR numeric NEXT abilit* OR reasoning NEXT abilit* OR “academic achievement” OR achievement NEXT score* OR “academic success” OR language NEXT score* OR reading NEXT score* OR “reading performance” OR math NEXT score* OR “math performance” OR mathematics NEXT score* OR “mathematics performance” OR academic NEXT grade* OR standardized test NEXT score*) AND

(child* OR “school children” OR student* OR kid* OR school-goer* OR “children in school” OR “school going children” OR schoolchildren OR adolescen* OR teenage* OR teen-age*)

## References

[1] Janssen I., LeBlanc A.G. (2010). Systematic review of the health benefits of physical activity and fitness in school-aged children and youth. Int. J. Behav. Nutr. Phys. Act..

[2] Sibley B.A., Etnier J.L. (2003). The relationship between physical activity and cognition in children: A meta-analysis. Pediatr. Exerc. Sci..

[3] Fedewa A.L., Ahn S. (2011). The effects of physical activity and physical fitness on children’s achievement and cognitive outcomes: A meta-analysis. Res. Q. Exerc. Sport.

[4] Donnelly J.E., Hillman C.H., Castelli D., Etnier J.L., Lee S., Tomporowski P., Lambourne K., Szabo-Reed A.N. (2016). Physical activity, fitness, cognitive function, and academic achievement in children: A systematic review. Med. Sci. Sports Exerc..

[5] Davis C.L., Tomporowski P.D., McDowell J.E., Austin B.P., Miller P.H., Yanasak N.E., Allison J.D., Naglieri J.A. (2011). Exercise improves executive function and achievement and alters brain activation in overweight children: A randomized, controlled trial. Health Psychol..

[6] Schaeffer D.J., Krafft C.E., Schwarz N.F., Chi L., Rodrigue A.L., Pierce J.E., Allison J.D., Yanasak N.E., Liu T., Davis C.L. (2014). An 8-month exercise intervention alters frontotemporal white matter integrity in overweight children. Psychophysiology.

[7] Hillman C.H., Pontifex M.B., Castelli D.M., Khan N.A., Raine L.B., Scudder M.R., Drollette E.S., Moore R.D., Wu C.-T., Kamijo K. (2014). Effects of the FITKids randomized controlled trial on executive control and brain function. Pediatrics.

[8] Khan N.A., Hillman C.H. (2014). The relation of childhood physical activity and aerobic fitness to brain function and cognition: A review. Pediatr. Exerc. Sci..

[9] Hallal P.C., Andersen L.B., Bull F.C., Guthold R., Haskell W., Ekelund U., Lancet Physical Activity Series Working Group (2012). Global physical activity levels: Surveillance progress, pitfalls, and prospects. Lancet.

[10] Cooper A.R., Goodman A., Page A.S., Sherar L.B., Esliger D.W., van Sluijs E.M., Andersen L.B., Anderssen S., Cardon G., Davey R. (2015). Objectively measured physical activity and sedentary time in youth: The International children’s accelerometry database (ICAD). Int J. Behav. Nutr. Phys. Act..

[11] Center for Disease Control and Prevention (2003). Physical activity levels among children aged 9–13 years—United States, 2002. MMWR Morb. Mortal. Wkly. Rep..

[12] Troiano R.P., Berrigan D., Dodd K.W., Masse L.C., Tilert T., McDowell M. (2008). Physical activity in the United States measured by accelerometer. Med. Sci. Sports Exerc..

[13] Migueles J.H., Cadenas-Sanchez C., Tudor-Locke C., Löf M., Esteban-Cornejo I., Molina-Garcia P., Mora-Gonzalez J., Rodriguez-Ayllon M., Garcia-Marmol E., Ekelund U. (2019). Comparability of published cut-points for the assessment of physical activity: Implications for data harmonization. Scand. J. Med. Sci. Sports.

[14] Tremblay M.S., Gray C.E., Akinroye K., Harrington D.M., Katzmarzyk P.T., Lambert E.V., Liukkonen J., Maddison R., Ocansey R.T., Onywera V.O. (2014). Physical activity of children: A global matrix of grades comparing 15 countries. J. Phys. Act. Health.

[15] Ogden C.L., Carroll M.D., Lawman H.G., Fryar C.D., Kruszon-Moran D., Kit B.K., Flegal K.M. (2016). Trends in Obesity Prevalence Among Children and Adolescents in the United States, 1988–1994 Through 2013–2014. JAMA.

[16] Tudor-Locke C., Ainsworth B.E., Popkin B.M. (2001). Active commuting to school. Sports Med..

[17] Davison K.K., Werder J.L., Lawson C.T. (2008). Peer reviewed: Children’s active commuting to school: Current knowledge and future directions. Prev. Chronic Dis..

[18] Chillón P., Evenson K.R., Vaughn A., Ward D.S. (2011). Systematic review of interventions for promoting active transportation to school. Int. J. Behav. Nutr. Phys. Act..

[19] Villa-Gonzalez E., Barranco-Ruiz Y., Evenson K.R., Chillón P. (2018). Systematic review of interventions for promoting active school transport. Prev. Med..

[20] Larouche R., Mammen G., Rowe D.A., Faulkner G. (2018). Effectiveness of active school transport interventions: A systematic review and update. BMC Public Health.

[21] Lee M.C., Orenstein M.R., Richardson M.J. (2008). Systematic review of active commuting to school and children’s physical activity and weight. J. Phys. Act. Health.

[22] Larouche R., Saunders T.J., John Faulkner G.E., Colley R., Tremblay M. (2014). Associations between active school transport and physical activity, body composition, and cardiovascular fitness: A systematic review of 68 studies. J. Phys. Act. Health.

[23] Faulkner G.E., Buliung R.N., Flora P.K., Fusco C. (2009). Active school transport, physical activity levels and body weight of children and youth: A systematic review. Prev. Med..

[24] Thompson Coon J., Boddy K., Stein K., Whear R., Barton J., Depledge M.H. (2011). Does participating in physical activity in outdoor natural environments have a greater effect on physical and mental wellbeing than physical activity indoors? A systematic review. Environ. Sci. Technol..

[25] Lubans D.R., Boreham C.A., Kelly P., Foster C.E. (2011). The relationship between active travel to school and health-related fitness in children and adolescents: A systematic review. Int. J. Behav. Nutr. Phys. Act..

[26] De Nazelle A., Nieuwenhuijsen M.J., Antó J.M., Brauer M., Briggs D., Braun-Fahrlander C., Cavill N., Cooper A.R., Desqueyroux H., Fruin S. (2011). Improving health through policies that promote active travel: A review of evidence to support integrated health impact assessment. Environ. Int..

[27] Moher D., Liberati A., Tetzlaff J., Altman D.G., Group P. (2009). Preferred reporting items for systematic reviews and meta-analyses: The PRISMA statement. PLoS Med..

[28] Mallen C., Peat G., Croft P. (2006). Quality assessment of observational studies is not commonplace in systematic reviews. J. Clin. Epidemiol..

[29] Hancock G.R., Mueller R.O., Stapleton L.M. (2010). The Reviewer’s Guide to Quantitative Methods in the Social Sciences.

[30] National Institutes of Health (2016). Quality Assessment Tool for Observational Cohort and Cross-Sectional Studies.

[31] Tercedor P., Villa-Gonzalez E., Avila-Garcia M., Diaz-Piedra C., Martinez-Baena A., Soriano-Maldonado A., Perez-Lopez I.J., Garcia-Rodriguez I., Mandic S., Palomares-Cuadros J. (2017). A school-based physical activity promotion intervention in children: Rationale and study protocol for the PREVIENE Project. BMC Public Health.

[32] Bugge A., Tarp J., Østergaard L., Domazet S.L., Andersen L.B., Froberg K. (2014). LCoMotion-Learning, Cognition and Motion; a multicomponent cluster randomized school-based intervention aimed at increasing learning and cognition—Rationale, design and methods. BMC Public Health.

[33] Tarp J., Domazet S.L., Froberg K., Hillman C.H., Andersen L.B., Bugge A. (2016). Effectiveness of a school-based physical activity intervention on cognitive performance in Danish adolescents: LCoMotion-Learning, cognition and motion—A cluster randomized controlled trial. PLoS ONE.

[34] Tigre R., Sampaio B., Menezes T. (2017). The impact of commuting time on youth’s school performance. J. Reg. Sci..

[35] Wright C.M., Duquesnay P.J., Anzman-Frasca S., Chomitz V.R., Chui K., Economos C.D., Langevin E.G., Nelson M.E., Sacheck J.M. (2016). Study protocol: The Fueling Learning through Exercise (FLEX) study—A randomized controlled trial of the impact of school-based physical activity programs on children’s physical activity, cognitive function, and academic achievement. BMC Public Health.

[36] Kalantari H.-A., Esmaeilzadeh S. (2016). Association between academic achievement and physical status including physical activity, aerobic and muscular fitness tests in adolescent boys. Environ. Health Prev. Med..

[37] Zhang Y., Zhang D., Jiang Y., Sun W., Wang Y., Chen W., Li S., Shi L., Shen X., Zhang J. (2015). Association between physical activity and teacher-reported academic performance among fifth-graders in Shanghai: A quantile regression. PLoS ONE.

[38] Lemieux M., Godin G. (2009). How well do cognitive and environmental variables predict active commuting?. Int. J. Behav. Nutr. Phys. Act..

[39] Heidari M., Borujeni M.B., Borujeni M.G., Shirvani M. (2017). Relationship of lifestyle with academic achievement in nursing students. J. Clin. Diagn. Res..

[40] Galván M., Uauy R., López-Rodríguez G., Kain J. (2014). Association between childhood obesity, cognitive development, physical fitness and social-emotional wellbeing in a transitional economy. Ann. Hum. Biol..

[41] ClinicalTrials.gov (2018). Acute Effects of Physical Activity on Memory, Cognitive Performance and Brain Activity. https://clinicaltrials.gov/show/nct03391505.

[42] Moran M.R., Eizenberg E., Plaut P. (2017). Getting to know a place: Built environment walkability and children’s spatial representation of their home-school (h-s) route. Int. J. Environ. Res. Public Health.

[43] Lopez-Vicente M., Forns J., Esnaola M., Suades-Gonzalez E., Alvarez-Pedrerol M., Robinson O., Julvez J., Garcia-Aymerich J., Sunyer J. (2016). Physical activity and cognitive trajectories in schoolchildren. Pediatr. Exerc. Sci..

[44] Haapala E.A., Poikkeus A.M., Kukkonen-Harjula K., Tompuri T., Lintu N., Vaisto J., Leppanen P.H.T., Laaksonen D.E., Lindi V., Lakka T.A. (2014). Associations of Physical Activity and Sedentary Behavior with Academic Skills—A Follow-Up Study among Primary School Children. PLoS ONE.

[45] Martinez-Gomez D., Ruiz J.R., Gomez-Martinez S., Chillon P., Rey-Lopez J.P., Diaz L.E., Castillo R., Veiga O.L., Marcos A., Avena Study G. (2011). Active Commuting to School and Cognitive Performance in Adolescents The AVENA Study. Arch. Pediatr. Adolesc. Med..

[46] Stea T.H., Torstveit M.K. (2014). Association of lifestyle habits and academic achievement in Norwegian adolescents: A cross-sectional study. BMC Public Health.

[47] Van Dijk M.L., De Groot R.H.M., Van Acker F., Savelberg H., Kirschner P.A. (2014). Active commuting to school, cognitive performance, and academic achievement: An observational study in Dutch adolescents using accelerometers. BMC Public Health.

[48] Domazet S.L., Tarp J., Huang T., Gejl A.K., Andersen L.B., Froberg K., Bugge A. (2016). Associations of physical activity, sports participation and active commuting on mathematic performance and inhibitory control in adolescents. PLoS ONE.

[49] Fang J.T., Lin J.J. (2017). School travel modes and children’s spatial cognition. Urban Stud..

[50] Garcia-Hermoso A., Saavedra J.M., Olloquequi J., Ramirez-Velez R. (2017). Associations between the duration of active commuting to school and academic achievement in rural Chilean adolescents. Environ. Health Prev. Med..

[51] Mora-Gonzalez J., Rodriguez-Lopez C., Cadenas-Sanchez C., Herrador-Colmenero M., Esteban-Cornejo I., Huertas-Delgado F.J., Ardoy D.N., Ortega F.B., Chillon P. (2017). Active commuting to school was inversely associated with academic achievement in primary but not secondary school students. Acta. Paediatr..

[52] Ruiz-Hermosa A., Martinez-Vizcaino V., Alvarez-Bueno C., Garcia-Prieto J.C., Pardo-Guijarro M.J., Sanchez-Lopez M. (2018). No Association Between Active Commuting to School, Adiposity, Fitness, and Cognition in Spanish Children: The MOVI-KIDS Study. J. Sch. Health..

[53] Westman J., Olsson L.E., Garling T., Friman M. (2017). Children’s travel to school: Satisfaction, current mood, and cognitive performance. Transportation.

[54] Tomporowski P.D., Davis C.L., Miller P.H., Naglieri J.A. (2008). Exercise and children’s intelligence, cognition, and academic achievement. Educ. Psychol. Rev..

[55] Giedd J.N., Blumenthal J., Jeffries N.O., Castellanos F.X., Liu H., Zijdenbos A., Paus T., Evans A.C., Rapoport J.L. (1999). Brain development during childhood and adolescence: A longitudinal MRI study. Nat. Neurosci..

[56] Moksnes U.K., Moljord I.E., Espnes G.A., Byrne D.G. (2010). The association between stress and emotional states in adolescents: The role of gender and self-esteem. Personal. Individ. Differ..

[57] Lupien S.J., McEwen B.S., Gunnar M.R., Heim C. (2009). Effects of stress throughout the lifespan on the brain, behaviour and cognition. Nat. Rev. Neurosci..

[58] Garcia-Segura L.M., Cardona-Gómez G.P., Chowen J.A., Azcoitia I. (2000). Insulin-like growth factor-I receptors and estrogen receptors interact in the promotion of neuronal survival and neuroprotection. J. Neurocytol..

[59] Frystyk J. (2010). Exercise and the growth hormone-insulin-like growth factor axis. Med. Sci. Sports Exerc..

[60] Herting M.M., Sowell E.R. (2017). Puberty and structural brain development in humans. Front. Neuroendocrinol..

[61] Klesges L.M., Baranowski T., Beech B., Cullen K., Murray D.M., Rochon J., Pratt C. (2004). Social desirability bias in self-reported dietary, physical activity and weight concerns measures in 8-to 10-year-old African-American girls: Results from the Girls Health Enrichment Multisite Studies (GEMS). Prev. Med..

[62] Dollman J., Okely A.D., Hardy L., Timperio A., Salmon J., Hills A.P. (2009). A hitchhiker’s guide to assessing young people’s physical activity: Deciding what method to use. J. Sci. Med. Sport.

[63] Jago R., Baranowski T., Baranowski J.C., Cullen K.W., Thompson D.I. (2006). Social desirability is associated with some physical activity, psychosocial variables and sedentary behavior but not self-reported physical activity among adolescent males. Health Educ. Res..

[64] Hillman C.H., Logan N.E., Shigeta T.T. (2019). A review of acute physical activity effects on brain and cognition in children. Transl. J. Am. Coll. Sports Med..

[65] Rovio S., Kåreholt I., Viitanen M., Winblad B., Tuomilehto J., Soininen H., Nissinen A., Kivipelto M. (2007). Work-related physical activity and the risk of dementia and Alzheimer’s disease. Int. J. Geriatr. Psychiatry.

[66] Ng T.P., Nyunt M.S.Z., Shuvo F.K., Eng J.Y., Yap K.B., Hee L.M., Chan S.P., Scherer S. (2018). The neighborhood built environment and cognitive function of older persons: Results from the Singapore Longitudinal Ageing Study. Gerontology.

[67] Carron A.V., Hausenblas H.A., Mack D. (2019). Social influence and exercise: A meta-analysis. J. Sport Exerc. Psychol..

[68] Clifford A., Lang L., Chen R., Anstey K.J., Seaton A. (2016). Exposure to air pollution and cognitive functioning across the life course—A systematic literature review. Environ. Res..

[69] Pujol J., Martínez-Vilavella G., Macià D., Fenoll R., Alvarez-Pedrerol M., Rivas I., Forns J., Blanco-Hinojo L., Capellades J., Querol X. (2016). Traffic pollution exposure is associated with altered brain connectivity in school children. Neuroimage.

[70] Lett L., Stingone J., Claudio L. (2017). The combined influence of air pollution and home learning environment on early cognitive skills in children. Int. J. Environ. Res. Public Health.

[71] Marcotte D.E. Something in the Air? Pollution, Allergens and Children’s Cognitive Functioning. https://ssrn.com/abstract=2725050.

[72] Alvarez-Pedrerol M., Rivas I., López-Vicente M., Suades-González E., Donaire-Gonzalez D., Cirach M., de Castro M., Esnaola M., Basagaña X., Dadvand P. (2017). Impact of commuting exposure to traffic-related air pollution on cognitive development in children walking to school. Environ. Pollut..

[73] Kerr J., Rosenberg D., Sallis J.F., Saelens B.E., Frank L.D., Conway T.L. (2006). Active commuting to school: Associations with environment and parental concerns. Med. Sci. Sports Exerc..

[74] McMillan T.E. (2007). The relative influence of urban form on a child’s travel mode to school. Transp. Res. Part A Policy Pract..

[75] Timperio A., Ball K., Salmon J., Roberts R., Giles-Corti B., Simmons D., Baur L.A., Crawford D. (2006). Personal, family, social, and environmental correlates of active commuting to school. Am. J. Prev. Med..

[76] Boarnet M.G., Anderson C.L., Day K., McMillan T., Alfonzo M. (2005). Evaluation of the California Safe Routes to School legislation: Urban form changes and children’s active transportation to school. Am. J. Prev. Med..

[77] Merom D., Tudor-Locke C., Bauman A., Rissel C. (2006). Active commuting to school among NSW primary school children: Implications for public health. Health Place.

[78] Diamond A. (2012). Activities and programs that improve children’s executive functions. Curr. Dir. Psychol. Sci..

